# Dimethyl­ammonium 4-hy­droxy­benzoate

**DOI:** 10.1107/S1600536812016145

**Published:** 2012-04-21

**Authors:** B. M. Sornamurthy, G. Peramaiyan, G. Chakkaravarthi, R. Mohan Kumar, V. Manivannan

**Affiliations:** a Department of Physics, Presidency College, Chennai 600 005, India; b Department of Physics, CPCL Polytechnic College, Chennai 600 068, India; c Department of Research and Development, PRIST University, Vallam, Thanjavur 613 403, Tamil Nadu, India

## Abstract

In the crystal structure of the title compound, C_2_H_8_N^+^·C_7_H_5_O_3_^−^, the anions and cations are linked by O—H⋯O and N—H⋯O hydrogen bonds into layers parallel to the *ac* plane.

## Related literature

For related structures, see: Hemamalini *et al.* (2011 ▶). Chitradevi *et al.* (2009 ▶). 
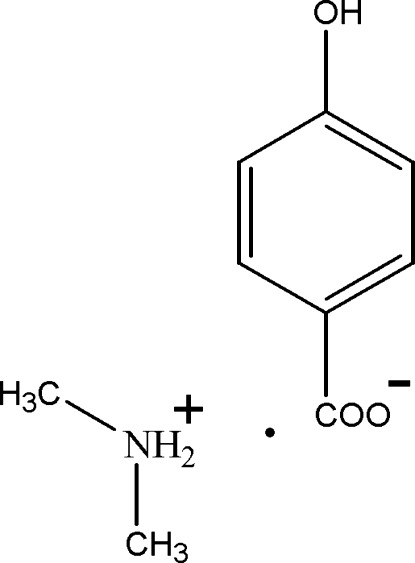


## Experimental

### 

#### Crystal data

C_2_H_8_N^+^·C_7_H_5_O_3_^−^
*M* *_r_* = 183.20
Orthorhombic, 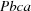

*a* = 10.2980 (8) Å
*b* = 10.0586 (9) Å
*c* = 19.2595 (17) Å
*V* = 1995.0 (3) Å^3^
*Z* = 8
Mo *K*α radiation
μ = 0.09 mm^−1^
*T* = 295 K
0.18 × 0.16 × 0.14 mm

#### Data collection

Bruker Kappa APEXII diffractometer
Absorption correction: multi-scan (*SADABS*; Sheldrick, 1996 ▶) *T*_min_ = 0.984, *T*_max_ = 0.987
9496 measured reflections
2394 independent reflections
1673 reflections with *I* > 2σ(*I*)
*R*_int_ = 0.036

#### Refinement

*R*[*F* ^2^ > 2σ(*F* ^2^)] = 0.047
*wR*(*F* ^2^) = 0.143
*S* = 1.04
2394 reflections
122 parameters
H-atom parameters constrained
Δρ_max_ = 0.29 e Å^−3^
Δρ_min_ = −0.27 e Å^−3^

### 

Data collection: *APEX2* (Bruker, 2004 ▶); cell refinement: *SAINT* (Bruker, 2004 ▶); data reduction: *SAINT*; program(s) used to solve structure: *SHELXS97* (Sheldrick, 2008 ▶); program(s) used to refine structure: *SHELXL97* (Sheldrick, 2008 ▶); molecular graphics: *PLATON* (Spek, 2009 ▶); software used to prepare material for publication: *SHELXL97*.

## Supplementary Material

Crystal structure: contains datablock(s) global, I. DOI: 10.1107/S1600536812016145/bt5869sup1.cif

Structure factors: contains datablock(s) I. DOI: 10.1107/S1600536812016145/bt5869Isup2.hkl

Supplementary material file. DOI: 10.1107/S1600536812016145/bt5869Isup3.cml

Additional supplementary materials:  crystallographic information; 3D view; checkCIF report

## Figures and Tables

**Table 1:** Hydrogen-bond geometry (Å, °)

*D*—H⋯*A*	*D*—H	H⋯*A*	*D*⋯*A*	*D*—H⋯*A*
N1—H1*A*⋯O3	0.90	1.87	2.7614 (18)	169
O1—H1⋯O2^i^	0.82	1.81	2.6183 (17)	171
N1—H1*B*⋯O2^ii^	0.90	1.82	2.7131 (17)	170

Symmetry codes: (i) 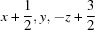; (ii) 
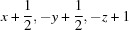
.

**Fig. 1.**
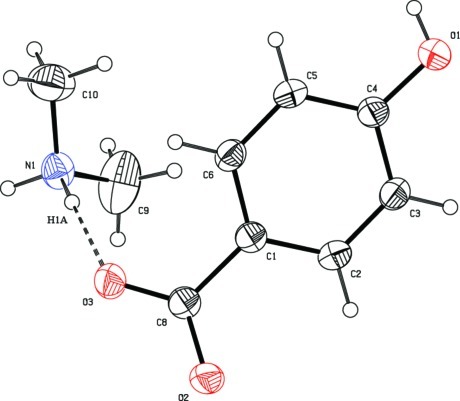
The molecular structure of (I), with atom labels and 30% probability displacement ellipsoids for non-H atoms.

**Fig. 2.**
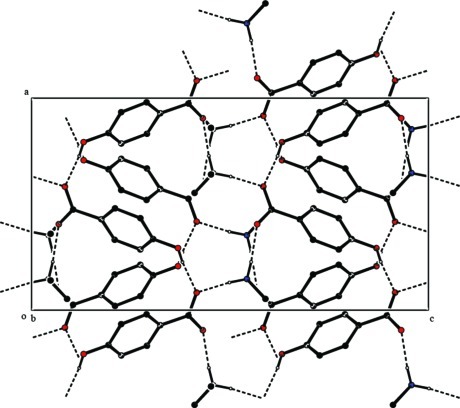
The packing of (I), viewed down b axis. Intermolecular Hydrogen bond is shown as dashed line. H atoms not involved in hydrogen bonding have been omitted.

## supplementary crystallographic information

### Comment

The goemetric parameters of the title compound (Fig. 1) are comparable with those in related structures (Hemamalini *et al.*, 2011; Chitradevi *et al.*, 2009).

The molecular structure is stabilized by intramolecular N—H···O hydrogen bond and the crystal structure is formed by weak intermolecular O—H···O and N-H···O (Fig. 2 & Table 1) interactions.

### Experimental

A solution of *p*-hydroxybenzoic acid (0.138g, 1 mmol) in 10 ml ethanol was added with stirring to a solution of dimethylamine (0.450g, 1 mmol) in 10 ml of distilled water at 303 K. After some time, a white precipitate was obtained. The white precipitate was dissolved in ethanol and colourless single crystals suitable for X-ray diffraction were obtained by slow evaporation of the ethanol solution.

### Refinement

All H atoms were located in a difference map, but positioned geometrically with O—H = 0.82Å, N—H = 0.90Å and C—H = 0.93–0.97 Å and allowed to ride on their parent atoms, with *U*_iso_(H) = 1.2U_eq_(C,N) or 1.5U_eq_(C_methyl_,O).

### Crystal data

**Table:**

C_2_H_8_N^+^·C_7_H_5_O_3_^−^	*F*(000) = 784
*M**_r_* = 183.20	*D*_x_ = 1.220 Mg m^−^^3^
Orthorhombic, *P**b**c**a*	Mo *K*α radiation, λ = 0.71073 Å
Hall symbol: -P 2ac 2ab	Cell parameters from 10382 reflections
*a* = 10.2980 (8) Å	θ = 2.1–28.2°
*b* = 10.0586 (9) Å	µ = 0.09 mm^−^^1^
*c* = 19.2595 (17) Å	*T* = 295 K
*V* = 1995.0 (3) Å^3^	Block, colourless
*Z* = 8	0.18 × 0.16 × 0.14 mm

### Data collection

**Table:**

Bruker Kappa APEXII diffractometer	2394 independent reflections
Radiation source: fine-focus sealed tube	1673 reflections with *I* > 2σ(*I*)
Graphite monochromator	*R*_int_ = 0.036
ω and φ scans	θ_max_ = 28.3°, θ_min_ = 2.1°
Absorption correction: multi-scan (*SADABS*; Sheldrick, 1996)	*h* = −13→12
*T*_min_ = 0.984, *T*_max_ = 0.987	*k* = −12→12
9496 measured reflections	*l* = −24→23

### Refinement

**Table:**

Refinement on *F*^2^	Secondary atom site location: difference Fourier map
Least-squares matrix: full	Hydrogen site location: inferred from neighbouring sites
*R*[*F*^2^ > 2σ(*F*^2^)] = 0.047	H-atom parameters constrained
*wR*(*F*^2^) = 0.143	*w* = 1/[σ^2^(*F*_o_^2^) + (0.0681*P*)^2^ + 0.4306*P*] where *P* = (*F*_o_^2^ + 2*F*_c_^2^)/3
*S* = 1.04	(Δ/σ)_max_ < 0.001
2394 reflections	Δρ_max_ = 0.29 e Å^−^^3^
122 parameters	Δρ_min_ = −0.27 e Å^−^^3^
0 restraints	Extinction correction: *SHELXL*, Fc^*^=kFc[1+0.001xFc^2^λ^3^/sin(2θ)]^-1/4^
Primary atom site location: structure-invariant direct methods	Extinction coefficient: 0.051 (4)

### Fractional atomic coordinates and isotropic or equivalent isotropic displacement parameters (Å^2^)

**Table:**

	*x*	*y*	*z*	*U*_iso_*/*U*_eq_	
C1	1.07130 (13)	0.17170 (15)	0.67576 (7)	0.0411 (4)	
C2	1.01976 (15)	0.06187 (15)	0.71025 (8)	0.0454 (4)	
H2	0.9527	0.0140	0.6897	0.055*	
C3	1.06624 (15)	0.02280 (16)	0.77417 (8)	0.0489 (4)	
H3	1.0315	−0.0518	0.7959	0.059*	
C4	1.16481 (14)	0.09434 (17)	0.80639 (8)	0.0463 (4)	
C5	1.21388 (15)	0.20718 (16)	0.77404 (8)	0.0479 (4)	
H5	1.2774	0.2579	0.7959	0.058*	
C6	1.16815 (14)	0.24402 (15)	0.70933 (8)	0.0453 (4)	
H6	1.2028	0.3187	0.6877	0.054*	
C8	1.02545 (14)	0.21186 (16)	0.60471 (8)	0.0446 (4)	
C9	1.3600 (3)	0.0899 (2)	0.54792 (15)	0.1060 (10)	
H9A	1.4478	0.0611	0.5406	0.159*	
H9B	1.3047	0.0507	0.5134	0.159*	
H9C	1.3318	0.0627	0.5933	0.159*	
C10	1.4471 (2)	0.3010 (3)	0.58929 (11)	0.0863 (7)	
H10A	1.4299	0.2755	0.6364	0.129*	
H10B	1.4385	0.3956	0.5848	0.129*	
H10C	1.5338	0.2750	0.5770	0.129*	
N1	1.35343 (14)	0.23451 (14)	0.54264 (7)	0.0525 (4)	
H1A	1.2726	0.2616	0.5533	0.063*	
H1B	1.3698	0.2589	0.4985	0.063*	
O1	1.20733 (12)	0.05085 (14)	0.86889 (6)	0.0650 (4)	
H1	1.2727	0.0922	0.8800	0.098*	
O2	0.91717 (11)	0.16686 (14)	0.58397 (6)	0.0603 (4)	
O3	1.09461 (11)	0.28636 (13)	0.56856 (6)	0.0590 (4)	

### Atomic displacement parameters (Å^2^)

**Table:**

	*U*^11^	*U*^22^	*U*^33^	*U*^12^	*U*^13^	*U*^23^
C1	0.0336 (7)	0.0454 (8)	0.0441 (8)	0.0053 (6)	0.0039 (6)	−0.0005 (6)
C2	0.0371 (8)	0.0468 (9)	0.0524 (9)	−0.0036 (6)	−0.0020 (6)	−0.0028 (7)
C3	0.0422 (8)	0.0487 (9)	0.0557 (9)	−0.0063 (7)	0.0003 (7)	0.0073 (7)
C4	0.0363 (8)	0.0561 (9)	0.0465 (8)	0.0000 (7)	0.0001 (6)	0.0050 (7)
C5	0.0386 (8)	0.0518 (9)	0.0534 (9)	−0.0072 (7)	−0.0047 (6)	−0.0014 (7)
C6	0.0383 (8)	0.0442 (8)	0.0533 (9)	−0.0022 (6)	0.0027 (6)	0.0034 (6)
C8	0.0349 (8)	0.0524 (9)	0.0467 (8)	0.0063 (6)	0.0049 (6)	0.0011 (7)
C9	0.130 (2)	0.0600 (14)	0.128 (2)	0.0119 (14)	0.0553 (19)	0.0005 (14)
C10	0.0676 (13)	0.125 (2)	0.0665 (13)	−0.0039 (13)	−0.0052 (11)	−0.0110 (13)
N1	0.0505 (8)	0.0579 (9)	0.0492 (8)	0.0060 (6)	0.0098 (6)	0.0028 (6)
O1	0.0531 (7)	0.0878 (10)	0.0541 (7)	−0.0161 (6)	−0.0109 (5)	0.0198 (6)
O2	0.0446 (7)	0.0909 (10)	0.0454 (6)	−0.0114 (6)	−0.0022 (5)	0.0078 (6)
O3	0.0462 (7)	0.0713 (8)	0.0596 (7)	−0.0017 (6)	0.0014 (5)	0.0190 (6)

### Geometric parameters (Å, º)

**Table:**

C1—C6	1.394 (2)	C8—O2	1.2680 (19)
C1—C2	1.394 (2)	C9—N1	1.460 (3)
C1—C8	1.503 (2)	C9—H9A	0.9600
C2—C3	1.378 (2)	C9—H9B	0.9600
C2—H2	0.9300	C9—H9C	0.9600
C3—C4	1.390 (2)	C10—N1	1.478 (3)
C3—H3	0.9300	C10—H10A	0.9600
C4—O1	1.3535 (19)	C10—H10B	0.9600
C4—C5	1.390 (2)	C10—H10C	0.9600
C5—C6	1.383 (2)	N1—H1A	0.9000
C5—H5	0.9300	N1—H1B	0.9000
C6—H6	0.9300	O1—H1	0.8200
C8—O3	1.2464 (19)		
			
C6—C1—C2	117.72 (14)	O2—C8—C1	117.85 (14)
C6—C1—C8	120.46 (14)	N1—C9—H9A	109.5
C2—C1—C8	121.82 (14)	N1—C9—H9B	109.5
C3—C2—C1	121.30 (14)	H9A—C9—H9B	109.5
C3—C2—H2	119.4	N1—C9—H9C	109.5
C1—C2—H2	119.4	H9A—C9—H9C	109.5
C2—C3—C4	120.30 (15)	H9B—C9—H9C	109.5
C2—C3—H3	119.8	N1—C10—H10A	109.5
C4—C3—H3	119.8	N1—C10—H10B	109.5
O1—C4—C5	123.02 (14)	H10A—C10—H10B	109.5
O1—C4—C3	117.76 (14)	N1—C10—H10C	109.5
C5—C4—C3	119.21 (14)	H10A—C10—H10C	109.5
C6—C5—C4	119.93 (14)	H10B—C10—H10C	109.5
C6—C5—H5	120.0	C9—N1—C10	112.2 (2)
C4—C5—H5	120.0	C9—N1—H1A	109.2
C5—C6—C1	121.47 (14)	C10—N1—H1A	109.2
C5—C6—H6	119.3	C9—N1—H1B	109.2
C1—C6—H6	119.3	C10—N1—H1B	109.2
O3—C8—O2	122.76 (15)	H1A—N1—H1B	107.9
O3—C8—C1	119.39 (14)	C4—O1—H1	109.5
			
C6—C1—C2—C3	−2.5 (2)	C4—C5—C6—C1	1.2 (2)
C8—C1—C2—C3	177.33 (14)	C2—C1—C6—C5	1.3 (2)
C1—C2—C3—C4	1.2 (2)	C8—C1—C6—C5	−178.54 (14)
C2—C3—C4—O1	−179.52 (15)	C6—C1—C8—O3	18.5 (2)
C2—C3—C4—C5	1.4 (2)	C2—C1—C8—O3	−161.34 (15)
O1—C4—C5—C6	178.38 (15)	C6—C1—C8—O2	−162.06 (15)
C3—C4—C5—C6	−2.5 (2)	C2—C1—C8—O2	18.2 (2)

### Hydrogen-bond geometry (Å, º)

**Table:**

*D*—H···*A*	*D*—H	H···*A*	*D*···*A*	*D*—H···*A*
N1—H1*A*···O3	0.90	1.87	2.7614 (18)	169
O1—H1···O2^i^	0.82	1.81	2.6183 (17)	171
N1—H1*B*···O2^ii^	0.90	1.82	2.7131 (17)	170

Symmetry codes: (i) *x*+1/2, *y*, −*z*+3/2; (ii) *x*+1/2, −*y*+1/2, −*z*+1.
